# Studies on PVA/MAA:EA polymer blend films modified by Fe^3+^ doping

**DOI:** 10.1186/s42649-025-00109-3

**Published:** 2025-07-07

**Authors:** Neeruganti Obularajugari Gopal, Mahammad Hussain Basha, Ojha Pravakar, Yedurupaka Madhav Kumar

**Affiliations:** 1 Department of Physics, Vikrama Simhapuri University, Nellore, 524324 India; 2 Department of Physics (S&H), N.B.KR. Institute of Science and Technology, Vidyanagar, 524413 India

**Keywords:** PVA/MAA: EA Polymer blend, Fe^3+^ doping, TGA, XRD, Optical band gap, EPR and electrical conductivity

## Abstract

Pristine and Fe (1.0, 2.0, 3.0, 4.0 and 5.0 mol%) doped Polyvinyl alcohol/Methacrylic Acid- Ethyl Acrylate (PVA/MAA:EA) polymer blend films were prepared by solution casting method and characterized by various techniques. TGA analysis validates the occurrence of three distinct weight loss steps attributed to removal of volatile substances from both the surface and interior of the material, as well as the decomposition of the polymer. X-ray diffraction (XRD) investigations confirm a decrease in crystallinity as the dopant concentration increases. Scanning electron microscope (SEM) micrographs show uniform morphology. Fourier transform infrared (FTIR) spectrum exhibits bands characteristic of stretching and bending vibrations of O–H, C–H, C = C and C–O groups and the changes in the spectrum with dopant concentration show the miscibility of dopant with the polymer blend. UV–visible absorption spectra demonstrate the decrease of optical band gap as the concentration of Fe rises and exhibit absorption bands corresponding to the transitions ^6^A_1g_ → ^4^A_1g_ and ^6^A_1g_ → ^4^T_2g_ of Fe^3+^ ions in distorted octahedral symmetry. Electron paramagnetic resonance (EPR) spectra exhibit resonance signals one around g = 2.12 attributed to Fe^3+^ ions in the distorted octahedral environment and the other signal around g = 6.8 due to Fe^3+^ ions in rhombic symmetry. The number of spins engaged in the resonance as a function of dopant concentration is determined through EPR analysis and the paramagnetic susceptibility is estimated. The conductivity of Fe^3+^ ions doped PVA/MAA:EA polymer blend films increases with an increase in Fe^3+^ concentration, which is explained in terms of an increase in the amorphous phase due to doping.

## Introduction

Polymer films, co-polymers and polymer blends have occupied the center stage of research in the domains of chemistry, physics and biophysics due to their low specific weight, good mechanical strength and their application in solid polymer electrolytes, high temperature coatings, bio sensors, strong electrodes for batteries, etc. (Raza et al. 2021; Li et al. 2022; Dhandapani et al. 2022, L. Liu et.al. 2023, R.Gayatri et al. 2024). With economically low cost, mixing individual polymers and copolymers is an attractive route to obtain the new polymeric materials with fascinating properties (D.P. Simunec et.al. 2023, M.M. Hosny et.al.^ 2024^). In recent times, there has been a significant surge in the investigation of polymer blends on their potential utilization in the domains of light-stable color filters, solar cells, and anti microbial films (A.Nag et al. 2024, M.M. Hosny et.al. 2024, Y. Imai et.al. 2024, P. Meedecha et.al. 2024). In this context, PVA/MAA:EA polymer blend has drawn researchers attention because of its good environmental stability, easy process ability and transparency (T.Siddaiah et al. 2018, and Ojha et al. 2021, T.Siddaiah et al. 2022).

By incorporating appropriate dopants into the polymers, copolymers, and their blends, it is possible to manipulate the properties such as thermal, optical, mechanical and electrical characteristics in a favorable manner, which enables them to emerge as the most promising choice for various technological applications (Eterigho-Ikelegbe et al. 2021, A. Dorigato et al. 2024, T. Lalire et al. 2024). One of the approaches that has been used by most researchers and scientists to improve certain specific properties of a polymer blend system for various technological applications is by doping suitable transition metal ions required (F.M.Ali. 2019, O. Pravakar et al. 2019, Arnab Mondal et.al. 2023, P. Preechakasedkit et.al. 2023, K.V.G. Raghavendra et.al. 2024, I.D. Anyaogu et.al. 2025). Transition metal ion doped polymer blend films represent a new class of organic materials for various applications as the doping significantly impacts the structural, optical, thermal, and electrical characteristics of polymer blend films. The primary objective of this research is to investigate the changes in thermal, optical, structural, morphological, magnetic, and electrical characteristics of PVA/MAA:EA polymer blend films by the incorporation of Fe^3+^ ions.

### Experimental Process

#### Material preparation

PVA/MAA:EA polymer blend films, pure and doped with various concentrations Fe^3+^ ions (1.0, 2.0, 3.0, 4.0, and 5.0 mol%) were prepared using the solution casting method at ambient temperature. The water-soluble polymers purchased from Merck Millipore India Ltd. include the Polyvinylalcohol (PVA), which has a molecular weight of 17,000, and the Methacrylic Acid—Ethyl Acrylate copolymer (1:1) dispersion with a mean relative molecular weight of approximately 250,000. These polymers are highly soluble in water. A solution of PVA blended with MAA:EA copolymer (PVA:MAAEA) in a 50:50 wt % concentration was prepared at ambient temperature utilizing double distilled water. A volume of five milliliters of PVA solution was combined with an equal volume of MAA: EA solution, followed by magnetic stirring for 10–12 h in order to achieve a uniform mixture. Solutions of FeSO_4_ with desired concentrations of 1.0, 2.0, 3.0, 4.0, and 5.0 mol %were prepared at room temperature by using double distilled water. Subsequently, these dopant solutions were added to the polymer blend mixture solution. The clear and homogeneous mixture obtained by magnetically stirring the blend solution for a duration of 3–4 h was then cast onto polypropylene dishes. Pure and Fe doped polymer blend films were obtained by slow evaporation of the solution in the dishes at room temperature.

#### Characterization

To examine the thermal stability of polymer blend films doped with transition metal ions, thermogravimetric analysis (TGA) was conducted in the presence of nitrogen flow from 30 to 600 ^0^C, at the heating rate of 10 ^0^C/min utilizing the TA Q 50 thermal analysis system from TA instruments, USA. Scanning electron micrographs were obtained from SEM model TM–3000 with scanning attachment to study the surface morphology of the samples. X-ray diffraction measurements were carried out using a Siemens D5000 diffractometer with Cu Kα radiation (λ = 1.5406 Å). Using a Perkin-Elmer FTIR spectrometer, FTIR spectra of these films were recorded over a wavenumber range from 500 – 4000 cm^−1^. The JASCO UV–VIS-NIR spectrophotometer (model- V.700) was utilized to record the UV–Vis absorption spectra of both pure and doped films at room temperature within the range of 300–1100 nm. EPR spectra of the prepared films were recorded at room temperature, using Bruker EPR spectrometer fitted with a Bruker TE102 cavity that operates at X-band frequency. The impedance measurements were carried out using a computer-controlled phase-sensitive multimeter (PSM 1700) at room temperature, within the frequency range of 1 Hz to 5 MHz. In order to perform the measurements, circular samples of 20 mm diameter are kept between two parallel conducting plates to form a parallel plate capacitor like setup.

## Results & discussion

### Thermogravimetric studies

**Fig. 1:**
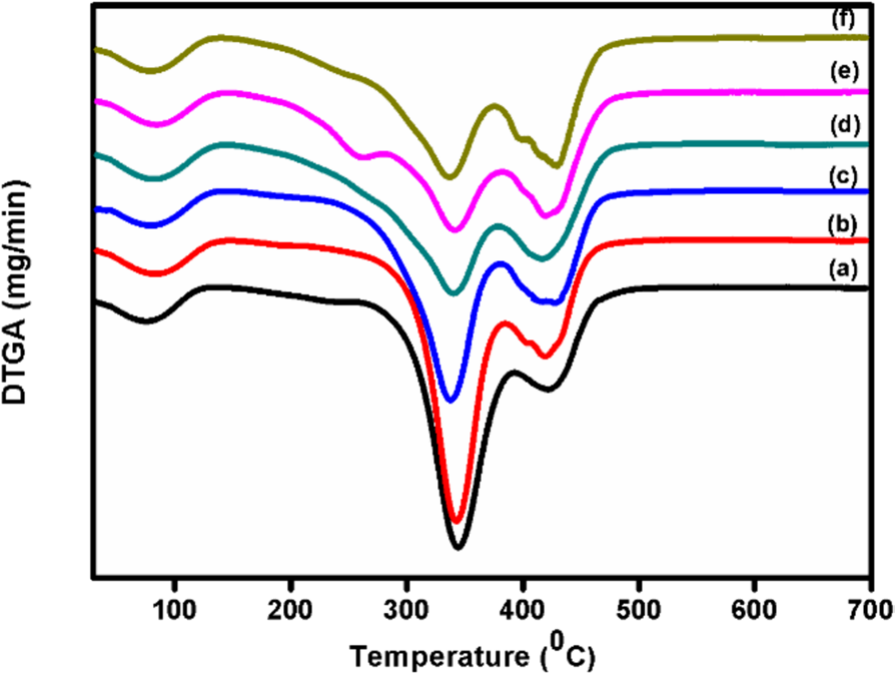
dTGAthermograms of (a) pure and different concentrations, (b) 1.0 (c) 2.0 (d) 3.0 (e) 4.0 and (f) 5.0 mol% of Fe^3+^doped PVA/MAA:EA polymer blend films

The dTGA thermograms of pure and different concentrations (1, 2, 3, 4 and 5 mol%) of Fe^3+^ ions doped PVA/MAA:EA polymer blend films are shown in Fig. 1. Analysis of dTGA curves reveal three distinct steps of degradation. The first degradation is observed from 35 to 144 ^0^C with a weight loss of 8% which is due to desorption of adsorbed water molecules in the samples (Tokizaki et al. 1991, N.H. Ahmad et.al. 2016). The second weight loss is observed in the temperature range 260–390 ^0^C, which includes the melting points and degradation temperatures of the polymer host (G.R. Mahdavinia et.al. 2014, Madhava Kumar et al. 2017a, b). The third stage of weight loss is observed in the range of 390–480 ^0^C, which may be attributed to the structural decomposition of the polymeric backbone (Guirguis et al. 2012, G. Asnag et.al. 2019). The thermal decompositions of all samples as well as percentages of weight loss were shown in the Table 1. It is observed that the value of weight loss was irregular for the second and third decomposition steps for all doped films with increasing dopant concentration. It is also observed that the peak temperature of the final decomposition step of pure and all doped samples is around 420 ^0^C. The value of total weight loss found to be decreasing with increase in dopant concentration from 1 to 5 mol%. Thus, it could be concluded that the thermal stability of PVA/MAA:EA polymer blend system increases with Fe^3+^ doping.

**Table 1:** dTGA data of weight loss steps and percentage of weight loss for pure and different concentrations (1.0, 2.0, 3.0, 4.0 and 5.0 mol%) of Fe^3+^ doped PVA/MAA:EA

Sample	Regions of weight loss	Temperature (^0^C)			Weight loss (%)	
		Start	End	T_p_	Partial	Total
	1st	35	135	85	8	
PVA/MAA:EA	2nd	262	390	343	37	90
	3rd	392	480	420	45	
	1st	35	144	93	8	
PVA/MAA:EA	2nd	262	391	342	36	84
1.0 mol% Fe	3rd	392	480	420	40	
	1st	37	142	87	8	
PVA/MAA:EA	2nd	262	391	342	35	80
2.0 mol% Fe	3rd	392	480	420	37	
	1st	38	144	88	8	
PVA/MAA:EA	2nd	262	392	344	34	78
3.0 mol% Fe	3rd	392	480	420	36	
	1st	35	134	88	8	
PVA/MAA:EA	2nd	236	305	275	15	
4.0 mol% Fe	3rd	305	392	368	20	75
	4th	392	480	420	32	
	1st	33	139	85	8	
PVA/MAA:EA	2nd	265	306	294	19	71
5.0 mol% Fe	3rd	306	392	348	11	
	4th	392	480	420	33	

### Studies on X-ray diffraction

**Fig. 2:**
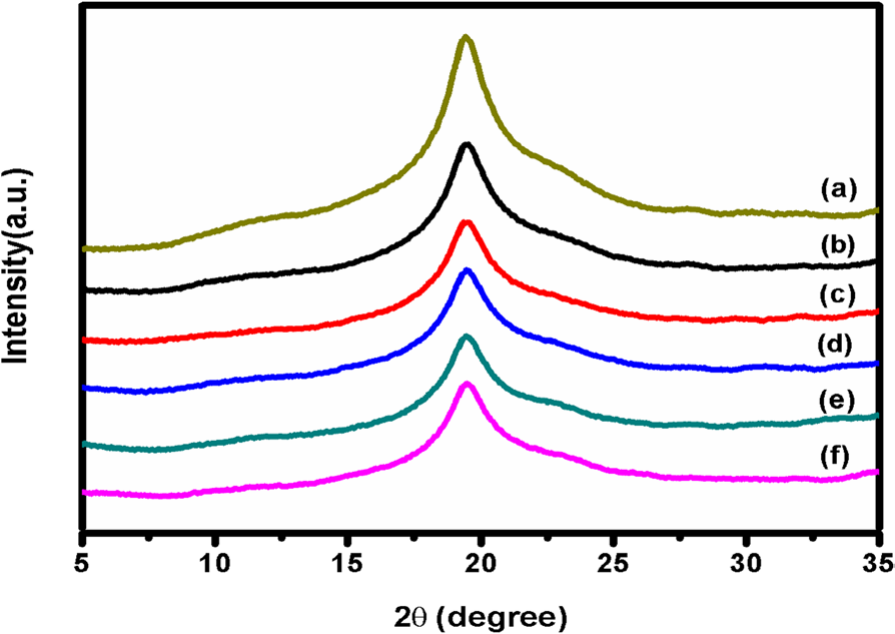
X-Ray diffraction (XRD) patterns obtained for (a) pure and various concentrations (b) 1.0 (c) 2.0 (d) 3.0 (e) 4.0 and (f) 5.0 mol% of iron (III) doped PVA/MAA:EA polymer blend films

The polymer matrix's complexation and crystallization can be determined by X-Ray diffraction analysis. Figure 2 displays the X-ray diffraction patterns of pure PVA/MAA:EA polymer blend film and the film doped with various concentrations of Fe^3+^.

As depicted by the XRD patterns, a single broad diffraction peak is observed at 2θ ≈ 20^0^ for pure and doped polymer blend films. This broad peak is known as the amorphous hump and is a typical characteristic of amorphous nature of the polymer (A. El Sayed et.al. 2015, M.A. Morsi et.al. 2019). For Fe doped samples, the broad nature of the diffraction peak persists but the intensity is decreased with increasing dopant concentration, which indicates a decrease in the crystallinity. This change in crystallization is because of strong intermolecular interaction between polymer blend and the dopant Fe due to free volume space offered by the blend for ion migration, which brings a change in the degree of crystallization (J.G. Durán-Guerrero et.al. 2018).

### Morphological studies

**Fig. 3:**
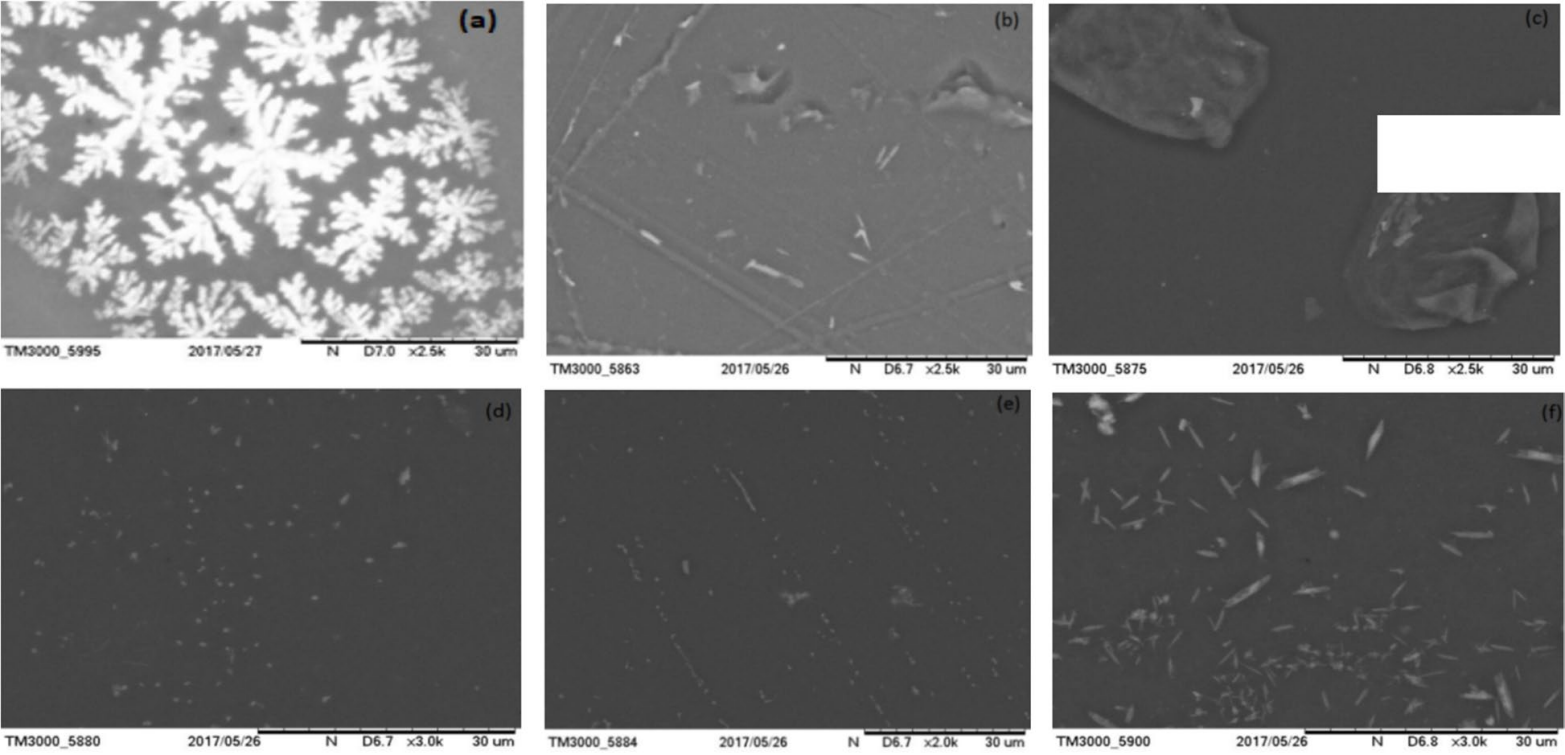
SEM images of (a) pure and different concentrations, (b) 1.0, (c) 2.0, (d) 3.0, (e) 4.0 and (f) 5.0 mol% of Fe^3+^ doped PVA/MAA:EA polymer blend films

SEM measurements were performed to examine fully the effect of Fe^3+^ dopant and the dispersion of Fe^3+^particles in the polymeric matrix and the micrographs are shown in Fig. 3. As shown in Fig. 3(a), the growth of the dendritic-like shape, which represents the gathering of the branched aggregate clusters, clearly indicates the formation of condensed aggregated dendrites shape. This suggests the presence of structural reorganizations of polymer chains (Sim et al. 2012). The surface morphology of the pure and Fe^3+^ (1.0, 2.0, 3.0, 4.0 and 5.0 mol%) doped PVA/MAA:EA polymer blend films is uniform, but with differing degrees of roughness (Fig. 3a–f). SEM micrographs suggest that the PVA/MAA:EA polymer blend molecules may disperse in the soft-segment phase with little influence on the micro phase separation and mixing of the hard and soft segments as shown in Fig. 3(a). After the addition of Fe^3+^ ions to pure PVA/MAA:EA polymer blend, it is observed that, there is an increase in the degree of roughness indicating the segregation of dopant in PVA/MAA:EA polymer blend film. This arises from random distribution and dissociation of dopant material which may introduce topological disorder in polymer blend film, which produces more amorphous phase in the system and makes the polymer film more flexible (Okerberg et al. 2008). The observed uniform surface morphology may be suitable for better conductivity of the doped polymer blend films (Noor et al. 2010).

### FTIR studies

**Fig. 4:**
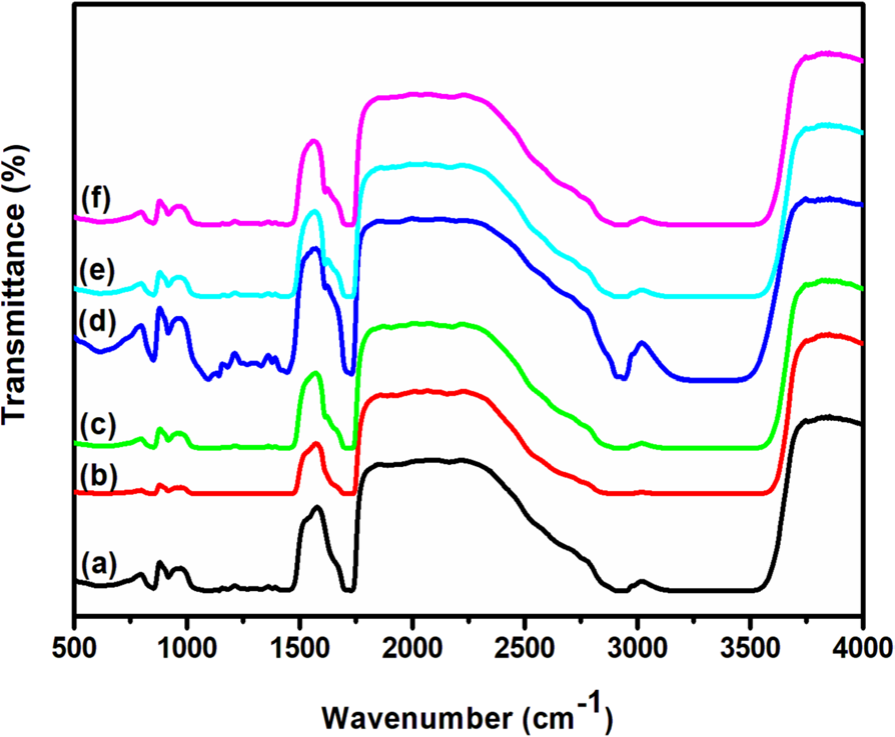
FTIR spectra of (a) pure and different concentrations, (b) 1.0, (c) 2.0, (d) 3.0, (e) 4.0and (f) 5.0 mol% of Fe^3+^ doped PVA/MAA:EA

FT-IR spectra of pure and doped PVA:MAA-EA films shown in Fig. 4 exhibit several bands characteristic of stretching and bending vibrations of O–H, C–H, C = C and C–O groups. For pure sample, the band observed at 3622 cm^−1^ is attributed to O–H stretching vibration. For doped samples, due to the presence of impurity ions, this peak shows shift in its position towards lower wavenumber and the shift is more prominent for the sample with 3 mol% Fe. The change in the band position indicates the coordination between –OH and Fe^3+^ (Saadiaha et al. 2019, Reddeppa et al.^ 2013^). Other vibrational bands do not show much variation in their position. The band observed at 2942 cm^−1^ indicates an asymmetry in stretching mode of CH_2_ group. A weak band observed at 2157 cm^−1^ is assigned to C = O group (Sreekanth et al. 2019). The band observed at 1741 cm^−1^ is assigned to stretching vibration of C = O group. A band observed at 1454 cm^−1^ corresponds to bending mode of vibration of CH_2_ group. The band observed at 1144 cm^−1^ corresponds to C–O stretching acetyl group present on the PVA backbone (Saadiaha et al.^ 2019^). The band observed at 918 cm^−1^ corresponds to stretching vibration C = C and a band at 856 cm^−1^ corresponds to stretching vibration of CH_2_ group (Vijaya Kumar et al. 2009). However, O–H stretching frequency observed at 3505 cm^−1^ for pure film exhibits appreciable shift towards low frequency region on doping Fe^3+^ions, which indicates the considerable interaction between O–H group of PVA and Fe^3+^ ions of FeSO_4_. The FTIR band positions and their assignments are given in the Table 2.

**Table 2:** Assignment of peak positions in FTIR spectrum of Fe^3+^ doped PVA/MAA:EA

Vibrational frequency (cm^−1^)	Band assignment
856	CH_2_(st)
918	C = C (st)
1144	C–O
1454	CH_2_(b)
1741	C = O(st)
2157	(C = O)
2942	CH_2_(st)
3505	OH(st)

### UV–Visible studies

**Fig. 5:**
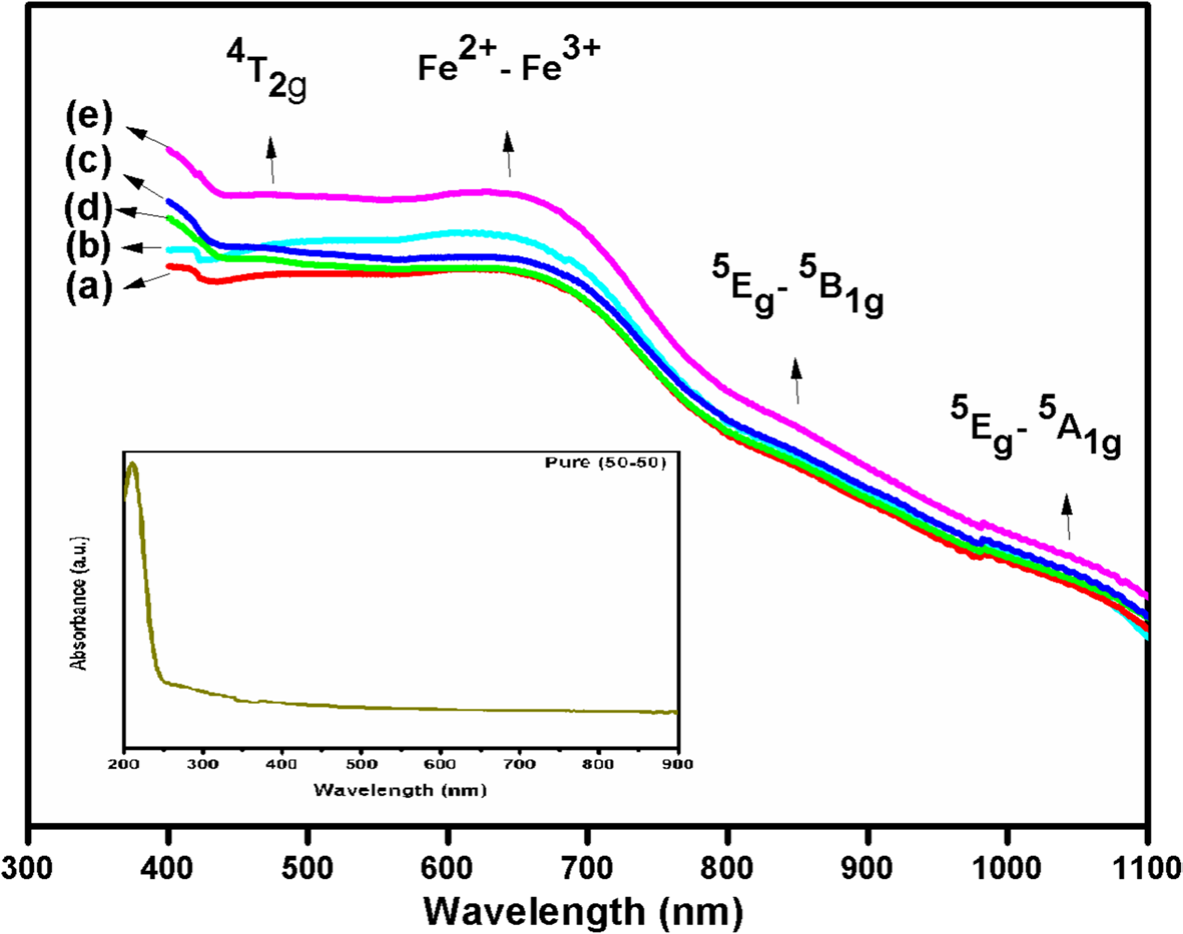
UV–Visible absorption spectra of different concentrations (a) 1.0, (b) 2.0, (c) 3.0, (d) 4.0 and (e) 5.0 mol% of Fe^3+^ doped PVA/MAA:EA polymer blend films

The optical absorption spectra of pure and Fe^3+^ (1.0, 2.0, 3.0, 4.0. and 5.0 mol%) doped PVA/MAA:EA polymer blend films recorded at room temperature in the wavelength range 300–1100 nm are shown in Fig. 5. The fundamental absorption observed in the spectra is used to determine the value of absorption edges and band gaps of the films to provide useful information about the band structure in both crystalline and non-crystalline state. The spectral changes observed can be interpreted in terms of the removal of an electron from the valance band and formation of polaron or bipolaron states upon doping (Abdelrazek et al. 2012). The peaks are considered to be due to the transitions from the valance band to the bipolaron states. The absorption spectrum for pure film is shown in the inset of Fig. 5.

The absorption bands observed at 402 and 460 nm have been assigned to d–d transitions ^6^A_1g_ → ^4^A_1g_ and ^6^A_1g_ → ^4^T_2g_ of Fe^3+^ ions respectively. In addition to these, a broad and intense band is also observed at 654 nm which may be attributed to the intervalence charge transfer (Fe^2+^– Fe^3+^) band (Sharma et al. 1991). However, it should be noted that the intervalence band transition peak does not change appreciably with increasing dopant concentration. The position of this peak is related to the degree of conjugation between the CH_3_ groups in the polymer chain. In the optical absorption spectrum of Fe^3+^ ions doped PVA/MAA:EA polymer blend films, two broad bands observed at 861and 1063 nm have been assigned to the spin-allowed d–d transitions ^5^E_g_ → ^5^B_1g_ and ^5^E_g_ → ^5^A_1g_ of Fe^2+^ ions in D_4h_ symmetry (Sharma et al. 1991).

**Fig. 6:**
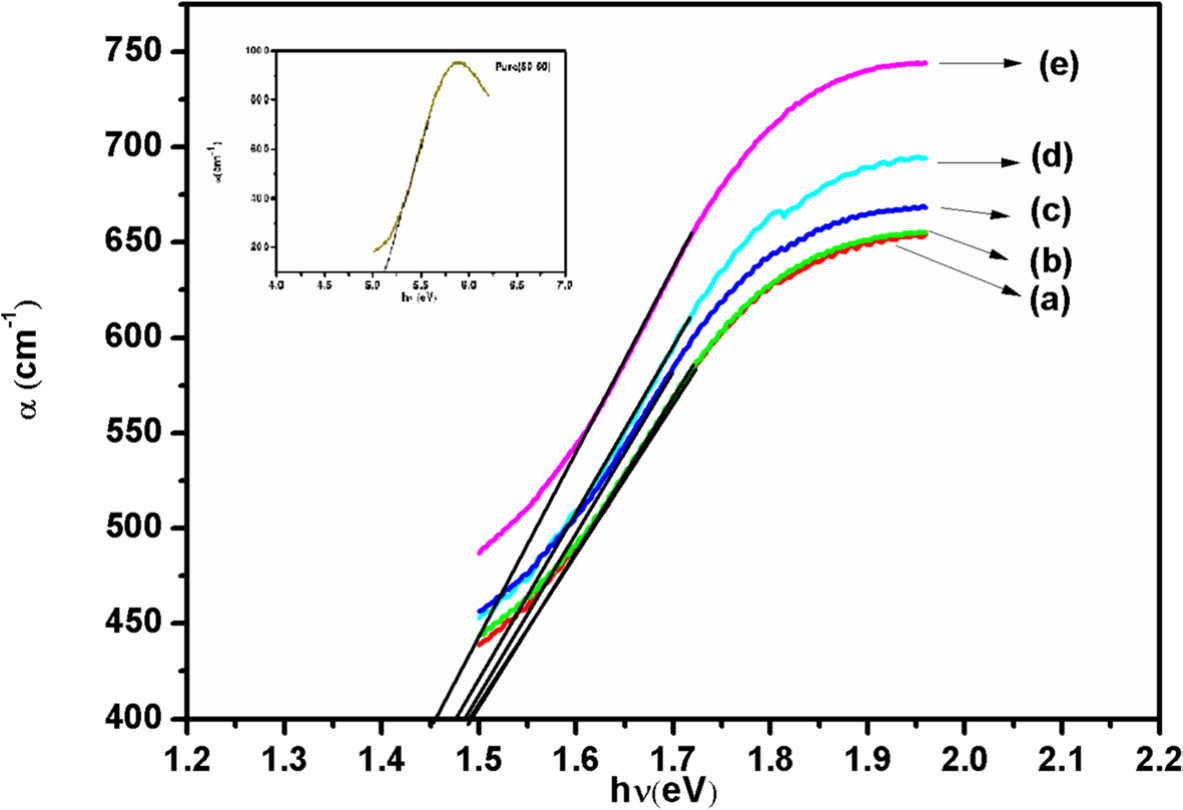
α vs hυ plots of different concentrations (a) 1.0, (b) 2.0, (c) 3.0, (d) 4.0 and (e) 5.0 mol% of Fe^3+^ doped PVA/MAA:EA polymer blend films

The absorption coefficient α is directly determined from the spectra by the formula (E.M. Abdelrazek et al. 2010),

1 $${\alpha }= 2.303 *(\text{A}/\text{d})$$

where ‘A’ indicates absorbance and ‘d’ thickness of the film. Figure 6 shows the variation in the absorption coefficient with incident photon energy for pure as well as Fe^3+^ doped PVA/MAA:EA polymer blend films. From the figure, it is clear that the absorption edge for pure film lies at 5.11 eV (shown in inset of Fig. 6) and for doped films the values are found to vary from 1.49 eV to 1.45 eV.

**Fig. 7:**
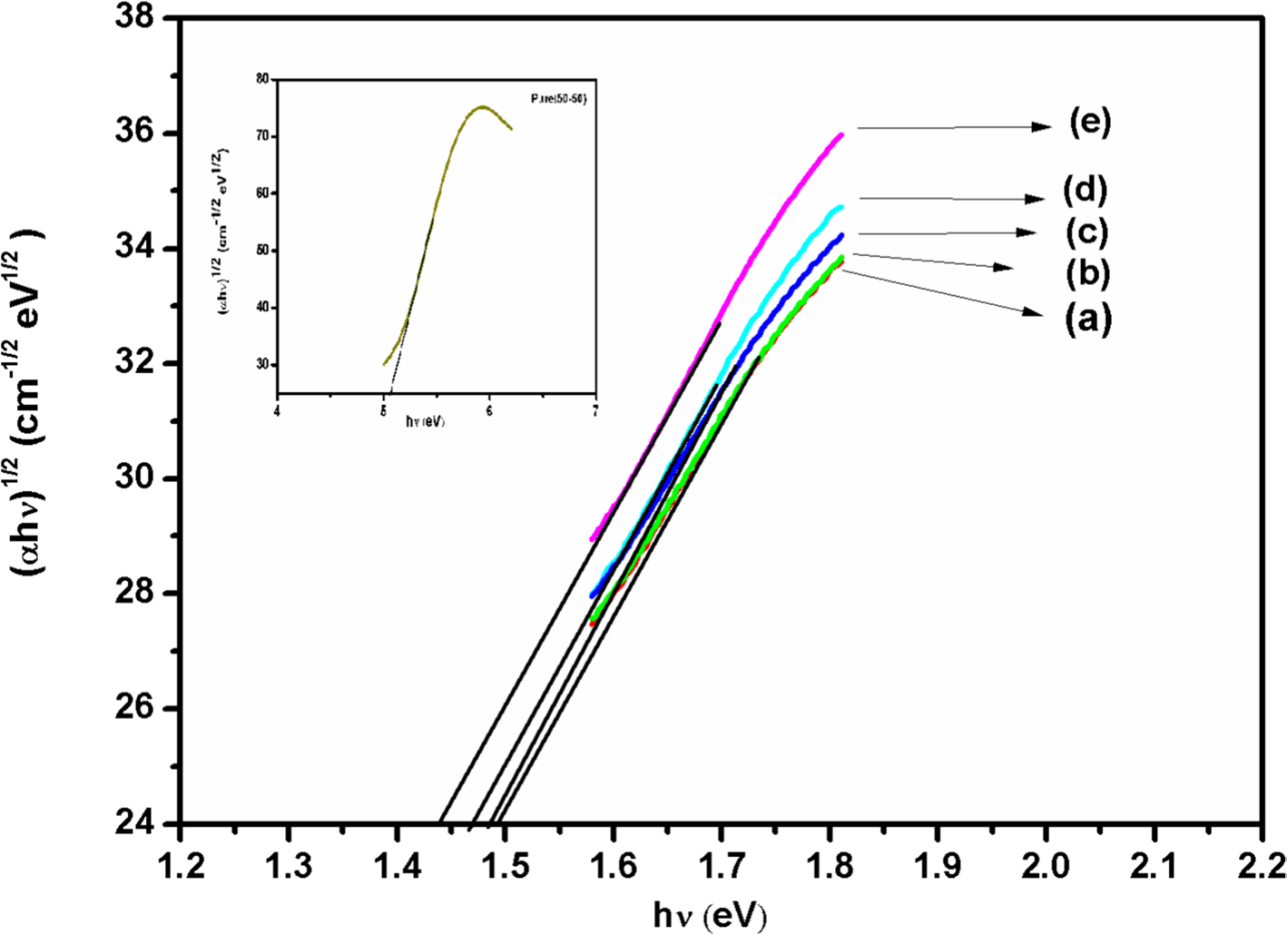
(αhυ)^1/2^ vs hυ plots of different concentrations (a) 1.0, (b) 2.0, (c) 3.0, (d) 4.0 and (e) 5.0 mol% of Fe^3+^ doped PVA/MAA:EA polymer blend films

In the indirect transitions, interaction occurs with lattice vibrations (phonons); so wave vector of the electron can change in the optical transition and momentum change will be taken or given by phonons. It means, when minimum of the conduction band lies in a different part of K-space from the maximum of the valence band, a direct optical transition from the top of the valence band to the bottom of the conduction band is forbidden. For indirect transition which requires phonon assistance, absorption coefficient has the following dependence on the photon energy (Madhava Kumar et al. 2017a, b, M. Morsi et al. 2019a, b, c).

2 $${\alpha h \nu }=\text{ B }({h\nu }-{{E}}_{\text{g}}+ {\text{E}}_{\text{p}}{)}^{2}+\text{ C }({h\nu }-{\text{E}}_{\text{g}}-{\text{E}}_{\text{p}}{)}^{2}$$

where ‘E_p_ represents phonon energy associated with transition and B, C are constants depending on the band structure. The indirect band gaps were obtained from the plots of (αhν)^1/2^ vs hν (Fig. 7). For pure PVA/MAA:EA polymer blend film, the indirect band gap lies at 5.09 eV (shown in inset of Fig. 7), while for doped films the values vary from 1.49 to 1.44 eV (Table 3). From Table 3, it is clear that the band edge and indirect band gap values decrease with the increase in dopant concentration. The decrease in optical band gap on doping may be explained on the basis of the fact that incorporation of small amount of dopants form charge transfer complexes in the host lattice. The band edge and indirect band gap values shifted to lower energies on doping with Fe^3+^ ions, this is due to the inter band transitions (A. El Sayed et al. 2015, G. Asnag et al 2019, A. Al-Ojeery 2023).

**Table 3:** Absorption edge and optical band gap values of pure and different concentrations of Fe^3+^ ions doped PVA/MAA:EA polymer blend films

Concentration in mol% Fe^3+^:PVA/MAA:EA	Absorption edge (eV)	Indirect band gap Energy (eV)
1.0 mol%	1.49	1.49
2.0 mol%	1.49	1.49
3.0 mol%	1.48	1.48
4.0 mol%	1.47	1.47
5.0 mol%	1.45	1.44

### EPR studies

**Fig. 8:**
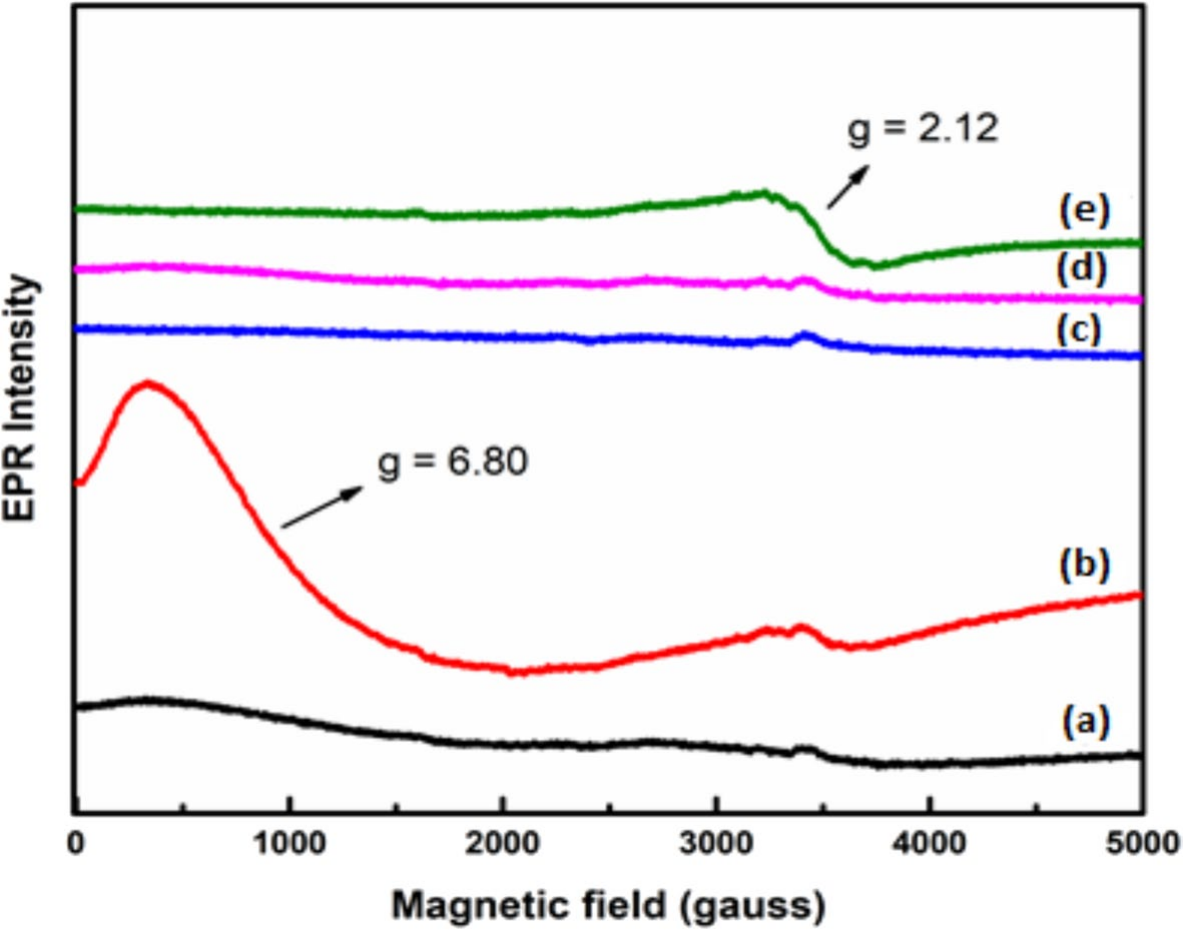
EPR spectra of (a) pure and different concentrations, (b) 1.0, (c) 2.0, (d) 3.0, (e) 4.0and (f) 5.0 mol% of Fe^3+^ doped PVA/MAA:EA

Electron Paramagnetic Resonance spectroscopy exposes the magnetic traits and spin dynamics, giving us a glimpse into the magnetic properties of materials and the inter particle dipolar interactions and super exchange interactions at play (Daruka Prasad et al. 2016, Ojha Pravakar et al. 2019). The spectra of the pure PVA/MAA:EA polymer blend film did not show any EPR signal, suggesting that the starting materials used in the present work were free from transition metal impurities or other paramagnetic centres. When various amounts of Fe^3+^ ions were doped to PVA/MAA:EA polymer blend, EPR spectra of all the investigated samples at room temperature shown in Fig. 8 exhibit two resonance signals one around g = 2.12 attributed to Fe^3+^ ions in the distorted octahedral environment and the other signal around g = 6.8 due to Fe^3+^ ions in rhombic symmetry (Daruka Prasad et al. 2016; Singh et al. 2011). When the concentration of Fe^3+^ ions is increased beyond 1 mol%, the signals around g = 6.8 has been disappeared and a broad signal has been observed at g = 2.12. This may due to the spin–spin interaction caused by the agglomeration of Fe^3+^ ions.

This variation suggests that the dopant affected the magnetic properties of the polymeric matrix. The evaluation of the EPR spectrum with 5 mol% depicts a decrease in the intensity of the deformed signal with respect to the 1 mol% spectrum. The broad signal can be attributed to the high concentration of Fe^3+^ ions. Moreover, the broad signal can be explained by the absence of isolated Fe^3+^ and the presence of Fe^3+^- Fe^3+^ exchange interaction leading to the existence of aggregated Fe^3+^ due to the proximity of iron ions. Consequently, the EPR demonstrations imply the formation of Fe^3+^ cluster in the polymeric matrix.

### Conductivity studies

**Fig. 9:**
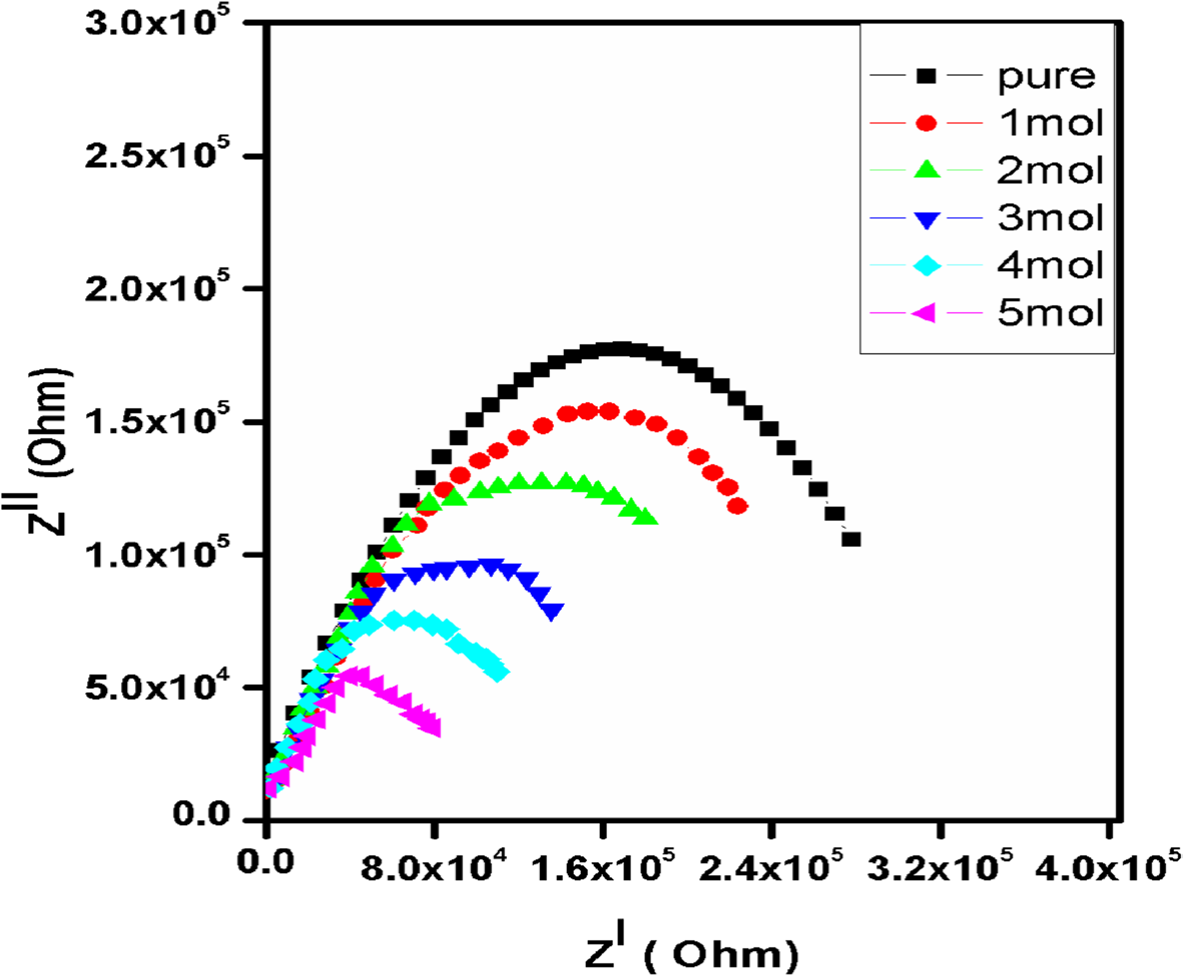
Nyquist impedance plots of pure and different concentrations of Fe^3+^ doped PVA/MAA:EA polymer blend films at room-temperature

Impedance spectroscopy is a versatile tool for ionic conductivity study of polymer blend films. Figure 9 shows the impedance plots for pure and different concentration of Fe^3+^ doped PVA/MAA:EA polymer blend films at room temperature in the frequency range of 1 Hz – 5 MHz. It is clear from Fig. 9 that, impedance plots of Z^׀׀^ as a function of Z^׀^, i.e. Cole – Cole plots of the films (Z^׀^ and Z^׀׀^ denote the real and imaginary parts of the complex impedance Z*) contain a semicircular arc, which is characteristic behavior of ionic conductivity of solids with blocking electrodes (Zidan et al.^ 2003^, M. Morsi et al 2019a, b, c, D.A. Nasrallah et al. 2022).

**Fig. 10:**
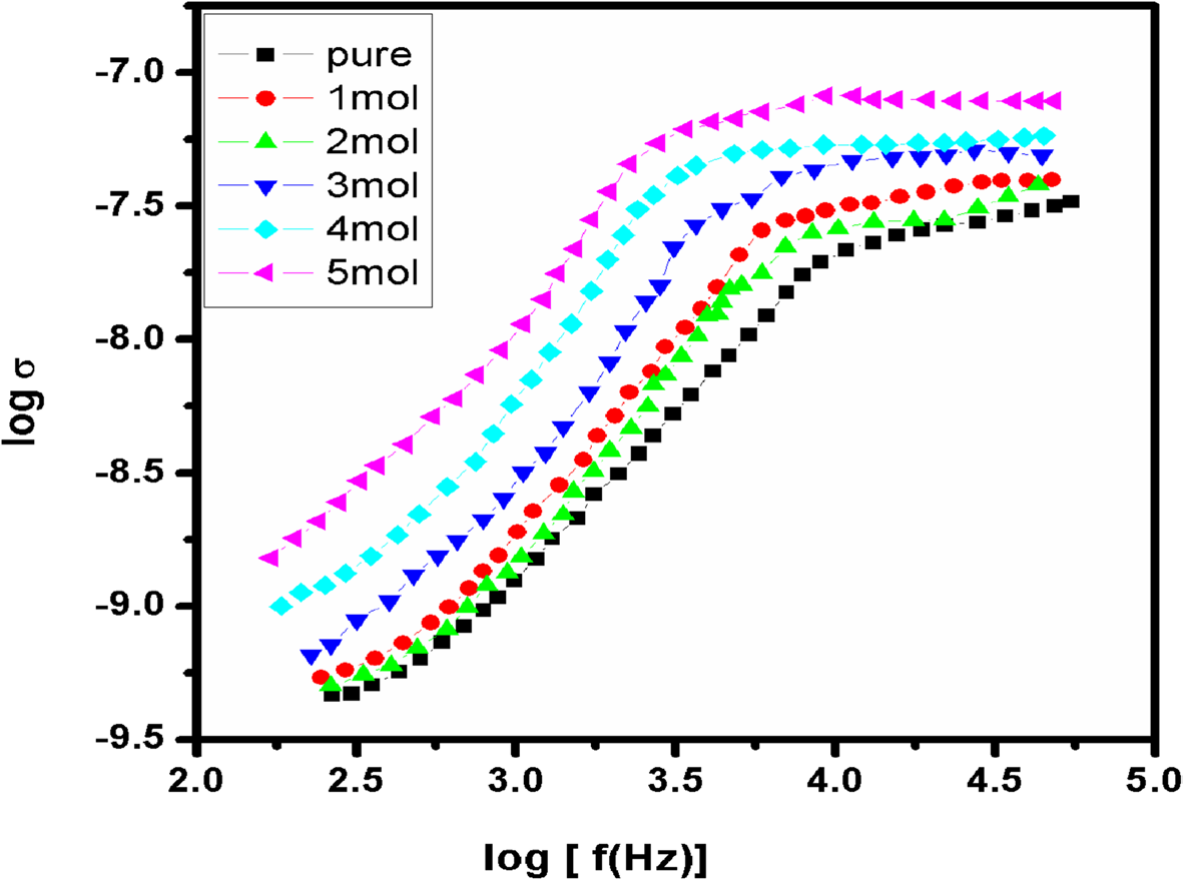
Variation of AC electrical conductivity (σ) of the doped samples with frequency of Fe^3+^ doped PVA/MAA:EA polymer blend films at room temperature

The semicircle shows the parallel combination between bulk resistance (due to the migration of ions) and bulk capacitance (due to the immobile polymer chains). Therefore frequency response of the sample could be represented by an equivalent circuit consisting of a parallel combination of the circuit elements R (resistance) and C (capacitance). The presence of the depressed semi circle reveals the non-Debye nature of the sample (Tawansi et al. 2004), due to the potential well for each site, through which the ion transport occurs. The inclined spike indicates a formation of double layer capacitance at the electrode–electrolyte interface as a result of migration of ions at low frequency. The capacitance values are in the range of PF, which represents the bulk response of the sample (Lanfredi et al. 2002). At each interface, electrode double layer possesses increasing impedance against ion transfer with the decrease in frequency, which, in the Nyquist plot of impedance spectra, was showed by an inclined spike. In addition, inclination of the spike at an angle less than 90^0^ to the real axis is due to the roughness of the electrode–electrolyte interface (Ramya et al. 2008). The ionic conductivity of pure and Fe^3+^ doped PVA/MAA:EA polymer blend films is calculated from the relation;

3 $$\sigma = l / {R}_{b}A$$

where ‘*l*’ is the thickness of the film, ‘A’ area of the film and ‘R_b_’ bulk resistance of the film material which is obtained from the intercept on the real axis at the high frequency end of the Nyquist plot of complex impedance (Macdonald et al. 1992). Figure 10 shows the variation of AC electrical conductivity of the doped samples with frequency at room temperature. Figure 10 shows existence of mobile charge carriers, which can be transported by hopping through defect sites along polymer blend chain (Saravanan et al. 2006). Also, there is an enhancement in the ionic conductivity of polymer blend films by adding Fe^3+^ ions, which is due to the increase in mobile charge carriers and charge carrier mobility, as well as due to an increase of amorphicity. This phenomena can be explained by a general conductivity relation;

4 $$\sigma = {n}_{i}{q}_{i}{\mu }_{i}$$

where $n_{i}$ is the number of charge carriers, $q_{i}$ is the charge of mobile charge carrier and $\mu_{i}$ is the mobility of charge carriers. According to this relation, the improvement in the ionic conductivity of polymer blend films can be achieved by increasing $n_{i}$ and $\mu_{i}$ because $q_{i}$ is the same for all charge carriers in the polymer blend system.

### Composition dependence of conductivity

**Fig. 11:**
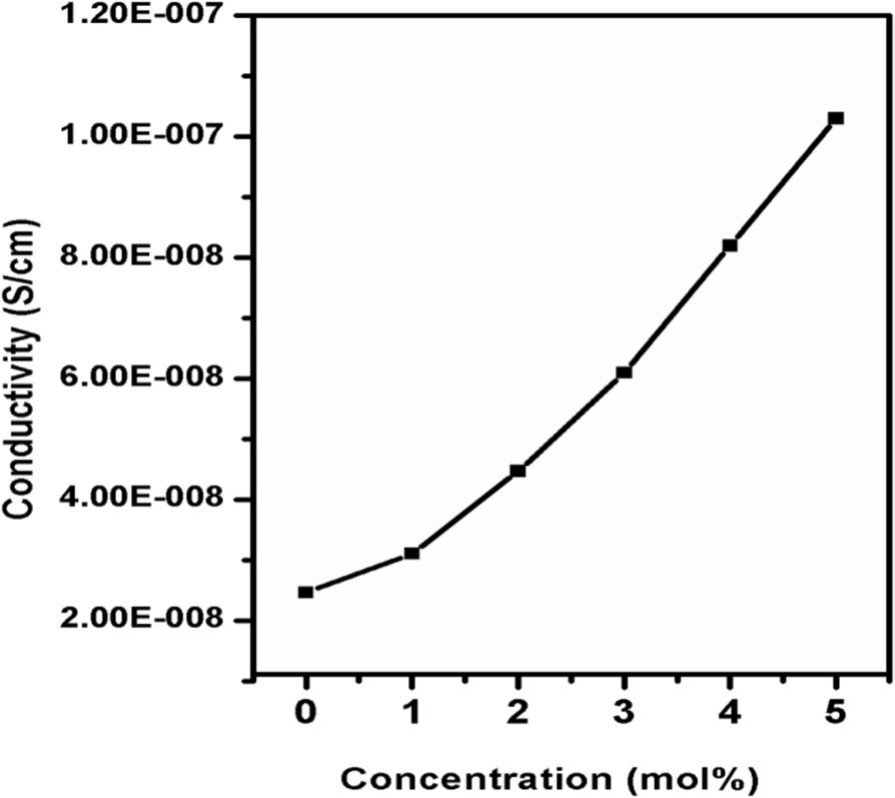
Variation of conductivity (σ) with different concentration of Fe^3+^ doped PVA/MAA:EA polymer blend films at room temperature

The variation of conductivity (σ) with Fe^3+^ concentration at room temperature is shown in Fig. 11. From the figure, it is noticed that the conductivity of pure film is about 2.4 X 10^–8^ Scm^−1^ at room temperature and increases to 1.03 X 10^–7^ Scm^−1^ for 5 mol% of Fe^3+^ ions doped films. The increase in ionic conductivity with increase in Fe^3+^ concentration is attributed to a reduction in crystallinity of polymer blend films and also to an increase in number of mobile charge carriers. The coordination interactions of ether oxygen atoms of PVA/MAA:EA polymer blend with Fe^3+^ cations, which result in a reduction in crystallinity of PVA/MAA:EA polymer blend, are responsible for the increase in ionic conductivity. The maximum conductivity shows the maximum and effective interaction between oxygen atoms and Fe^3+^ cations. A decrease in crystallinity of PVA/MAA:EA polymer blend is seen from the XRD analysis, whch indicates a reduction in the intensity of sharp crystalline peaks with the addition of Fe^3+^ ions, which results in a dominant amorphous phase in the polymer blend. A polymer chain in the amorphous phase is more flexible, which results in an increase in segmental motion of the polymer, which facilitates higher ionic mobility (Park et al. 2003). The increment in conductivity with increase in dopant concentration is due to the rise in the number of charge carriers as shown in Fig. 11. The conductivity data of pure and Fe^3+^ doped PVA/MAA:EA polymer blend at room temperature is presented in Table 4.

**Table 4:** Conductivity values of pure and different concentrations of Fe^3+^ doped PVA/MAA:EA polymer blend films at room temperature

Concentration in mol% of Fe^3+^:PVA/MAA:EA	Conductivity at 303 K (S cm^−1^)
Pure	2.46 × 10^–8^
1	3.11 × 10^–8^
2	4.47 × 10^–8^
3	6.1 × 10^–8^
4	8.2 × 10^–8^
5	1.03 × 10^–7^

## Conclusions

Pure and Fe doped PVA/MAA:EA polymer blend films were prepared by solution casting method and characterized by various techniques. The derivative thermogravimetric curves of the pure and Fe^3+^ doped PVA/MAA:EA polymer blend films show three distinct steps of weight loss, where first step is due to evaporation of adsorbed water, second step is due to loss of absorbed water from the bulk of the material, melting points & degradation of the large chain into small fragments and the third step is due to main chain decomposition. Amorphicity of the polymer blend films increases with increase of Fe^3+^ concentration as observed in the decrease in total weight loss for 1 to 5 mol%. X-ray diffraction patterns indicate an enhanced amorphous nature of the polymer electrolyte with increase in dopant concentration as observed by the decrease in the intensity of diffraction patterns. SEM micrographs show uniform morphology. The FTIR spectra exhibit bands characteristic of stretching, bending vibrations of O – H, C – H, C = O, CH_3_ – O and CH_2_ + α – CH_3_ groups. Optical absorption spectra showed that the absorption edge and indirect band gap values shifted to lower energies on doping with Fe^3+^ ions, thus indicating more semiconducting nature with increase in concentration of Fe^3+^. EPR spectra of all doped samples exhibit resonance signals one around g = 2.12 attributed to Fe^3+^ ions in the distorted octahedral environment and the other signal around g = 6.8 due to Fe^3+^ ions in rhombic symmetry. When the concentration of Fe^3+^ ions is increased beyond 1 mol%, the signals around g = 6.8 has been disappeared and a broad signal has been observed at g = 2.12, which may due to the spin–spin interaction caused by the agglomeration of Fe^3+^ ions. The conductivity of Fe^3+^ ions doped PVA/MAA:EA polymer blend films observed to increase with an increase in Fe^3+^ concentration, which is explained in terms of an increase in the amorphicity. Thus, these changes shown a high sensitivity of these films to doping that would suggest the applicability in magnetic and/or optical devices, fabricating solid state batteries and other electrochemical devices.

## Data Availability

The data that supports the findings of this study is available from the corresponding author upon reasonable request.

## Abbreviations

PVA/MAA:EA: Polyvinyl Alcohol/Methacrylic Acid-Ethyl Acrylate
TGA: Thermal Gravimetric Analysis
XRD: X-Ray Diffraction
SEM: Scanning Electron Microscope
FTIR: Fourier Transform Infrared Spectroscopy
EPR: Electron Paramagnetic Resonance
